# CD82 protects against glaucomatous axonal transport deficits via mTORC1 activation in mice

**DOI:** 10.1038/s41419-021-04445-6

**Published:** 2021-12-11

**Authors:** Meng Ye, Jingqiu Huang, Qianxue Mou, Jing Luo, Yuanyuan Hu, Xiaotong Lou, Ke Yao, Bowen Zhao, Qiming Duan, Xing Li, Hong Zhang, Yin Zhao

**Affiliations:** 1 Department of Ophthalmology, Tongji Hospital, Tongji Medical College, Huazhong University of Science and Technology, Wuhan, 430030 China; 2 Institute of Reproductive Health, Center for Reproductive Medicine, Tongji Medical College, Huazhong University of Science and Technology, Wuhan, China; 3 Gladstone Institutes, San Francisco, CA USA; 4 Department of Anesthesiology, Tongji Hospital, Tongji Medical College, Huazhong University of Science and Technology, Wuhan, 430030 China

**Keywords:** Molecular neuroscience, Neurodegeneration

## Abstract

Glaucoma is a leading cause of irreversible blindness worldwide and is characterized by progressive optic nerve degeneration and retinal ganglion cell loss. Axonal transport deficits have been demonstrated to be the earliest crucial pathophysiological changes underlying axonal degeneration in glaucoma. Here, we explored the role of the tetraspanin superfamily member CD82 in an acute ocular hypertension model. We found a transient downregulation of CD82 after acute IOP elevation, with parallel emergence of axonal transport deficits. The overexpression of CD82 with an AAV2/9 vector in the mouse retina improved optic nerve axonal transport and ameliorated subsequent axon degeneration. Moreover, the CD82 overexpression stimulated optic nerve regeneration and restored vision in a mouse optic nerve crush model. CD82 exerted a protective effect through the upregulation of TRAF2, which is an E3 ubiquitin ligase, and activated mTORC1 through K63-linked ubiquitylation and intracellular repositioning of Raptor. Therefore, our study offers deeper insight into the tetraspanin superfamily and demonstrates a potential neuroprotective strategy in glaucoma treatment.

## Introduction

Glaucoma is a neurodegenerative disease characterized by the progressive loss of retinal ganglion cells (RGCs) and visual fields [1]. Increased intraocular pressure (IOP) is one of the most important risk factors for glaucoma. Although RGC apoptosis is undoubtedly critical for disease development, current studies investigating glaucoma tend to deemphasize RGC death, which is known to appear late in the disease course, and pay more attention to early axonal lesions [2]. Axonal transport is important for maintaining proper neuronal function in the visual pathway since RGCs are highly polarized and have long axons. Axon transport impairments have been identified as the earliest crucial pathophysiological changes underlying axonal degeneration in glaucoma [3] and other neurodegenerative diseases [4–7]. It has been proposed that in eyes with increased IOP, the pressure difference across the optic nerve head (ONH) increases, compressing the optic nerve and, thus, impeding antero- and retrograde axonal transport [8, 9]. However, the critical feature of this process and the significance of early intervention remain to be further explored.

CD82 is a membrane glycoprotein belonging to the tetraspanin superfamily. The tetraspanin family is evolutionarily conserved and regulates the immune system, tumors, and cell proliferation [10]. Several tetraspanins also play critical roles in neuronal signaling and retinal degeneration [11–14]. CD82 is ubiquitously expressed in human tissues and is well known as a tumor suppressor protein [15]. Its effects on the neuronal system, specifically in the retina, have not yet been characterized.

The mechanistic target of rapamycin (mTOR) signaling pathway plays a vital role in cell growth, metabolism, and neuronal function [16]. Dysregulation of the mTOR pathway is involved in neurodegenerative diseases, such as Alzheimer’s disease, Parkinson’s disease, and glaucoma [17]. Several studies suggest that the mTOR pathway plays a role in maintaining the proper structure and function of RGCs [18–20]. Immunoprecipitation studies have revealed the expression of mTOR components in the inner retina, with mTORC1 predominantly localized in RGCs and their axons and, thus, directly involved in regulating RGC axonal function [21].

The proteins of the mTOR pathway can also interact with a subset of tetraspanins. [22, 23]. It has also been reported that CD82 overexpression in multiple myeloma cell lines resulted in the upregulation of phospho-S6 ribosomal protein, an established target of mTORC1 [24]. However, the specific mechanism underlying such regulation remains unclear.

TRAF2 is a pivotal member of the tumor necrosis factor (TNF) receptor-associated factor (TRAF) family, has E3 ubiquitin ligase activity, and possesses the ability to regulate multiple downstream signaling pathways by catalyzing linkages of polyubiquitin chains to specific substrates [25–29]. It has been reported that K63-linked ubiquitination is involved in the regulation of mTORC1 activity through the ubiquitin modification of different components and recruitment to lysosomes for further activation [30–32].

In the present study, we investigated the roles of CD82 in axonal transport deficits and degeneration using a mouse model of acute ocular hypertension (AOHT). We found that CD82 exerted a neuroprotective effect by activating the mTOR pathway via the intermediate molecule TRAF2. Our findings revealed a novel neuroprotective mechanism and provide a therapeutic target for glaucoma treatment.

## Results

### CD82 expression was downregulated in the retina, ONH, and ON in the early stage after the establishment of an acute ocular hypertension (AOHT) model

**Fig. 1:**
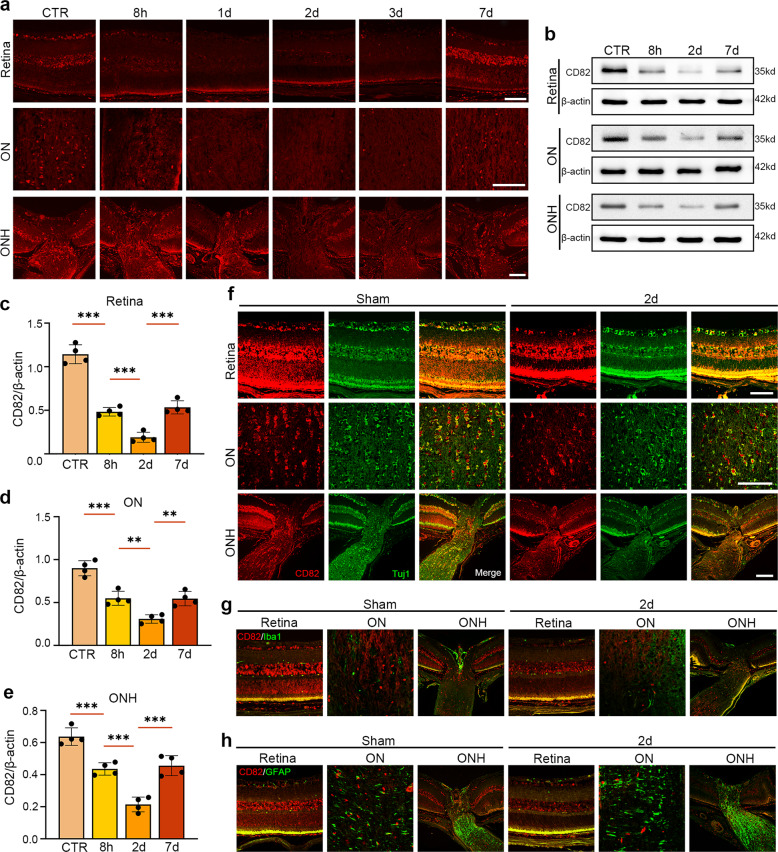
CD82 expression was down-regulated in retina, ONH, and ON in the early stage after acute IOP hypertension. **a** Immunostaining of CD82 in mouse retina, ON, ONH at 8 h, 1d, 2d, 3d, 7d after inducing AOHT, *n* = 5 eyes for each group. Scale bar, 50 μm. **b** Western blot showing protein levels of CD82 in mouse retina, ON, and ONH tissues at 8 h, 2d, 7d after AOHT. **c**−**e** Statistical analysis of the data shown in (**b**) from retina (**c**), ON (**d**), and ONH (**e**). The data were expressed as mean ± S.E.M., *n* = 4, one-way ANOVA **P < 0.01, ***P < 0.001. Co-immunofluorescent staining of CD82 (red) with RGC marker Tuj1 (green) (**f**), microglia marker Iba1 (green) (**g**), astrocyte marker (GFAP) (**h**) in mouse retina, ON, ONH in sham and 2d post-AOHT groups, *n* = 4 eyes for each group. Scale bars, 50 μm.

The protein levels of CD82 in the retina, unmyelinated optic nerve head (ONH), and posterior myelinated optic nerve (ON) were detected at different timepoints after AOHT. As shown in Fig. 1a, the results were similar across all three sites. CD82 immunofluorescence started to decline as early as 8 h, reached its lowest level on Day 2, and partially recovered on Day 7. The western blot analysis of the CD82 protein levels were consistent with the immunofluorescence staining (Fig. 1b−e).

Subsequently, we explored the cellular localization of CD82 in the posterior segment of the eye. Immunofluorescent double-labeling of CD82 with cell markers of RGCs (Tuj1), microglia (Iba1), and astrocytes (GAFP) was performed (Fig. 1f−h). It was apparent that CD82 was mainly localized parallel to the RGC marker Tuj1 but rarely colabeled with Iba1 or GAFP regardless of modeling, suggesting that CD82 mainly functions in RGCs and their axons rather than in other glial cells.

### CD82 overexpression in the retina and ON was achieved by intravitreal injection of recombinant adeno-associated virus rAAV2/9-Cd82-mCherry

**Fig. 2:**
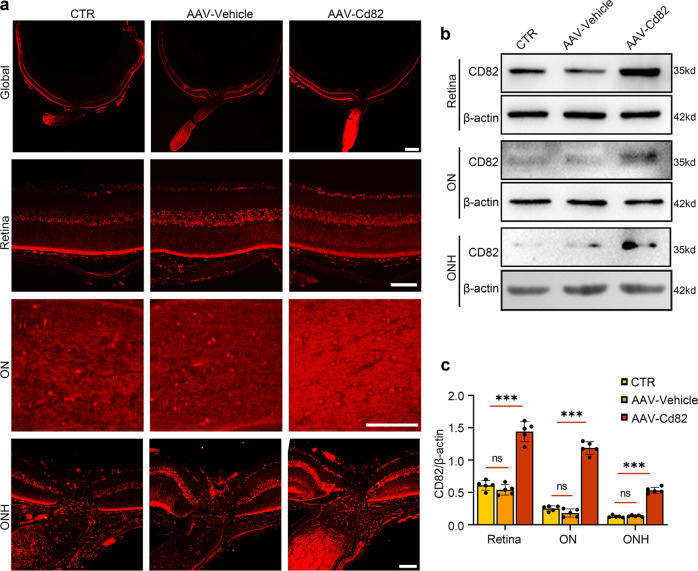
CD82 overexpression in retina and ON was achieved by Intravitreal injection of recombinant adeno-associated virus rAAV2/9-Cd82-mCherry. **a** Immunostaining of CD82 shown in whole globe, retina, ON, and ONH in CTR, AAV-Vehicle, AAV-Cd82 groups 28 days after virus injection, *n* = 5 eyes. Scale bars, 200 μm for Global; 50 μm for Retina, ON, and ONH. **b** Western blot showing protein levels of CD82 in mouse retina, ON and ONH tissues from CTR, AAV-Vehicle, AAV-Cd82 groups 28 days after virus injection. **c** Statistical analysis of the data shown in (**b**). The data were expressed as mean ± S.E.M., *n* = 5, one-way ANOVA ***P < 0.001.

To investigate the role of CD82 in the ocular hypertension mouse model, an adeno-associated virus vector was designed to overexpress CD82 in the mouse retina. rAAV2/9-Cd82-mCherry or rAAV2/9-mCherry (vehicle) was intravitreally injected 28 days before the mice were sacrificed, and the CD82 protein overexpression efficiency was verified by immunofluorescence and western blot analysis. It was evident that the CD82 immunofluorescence intensity in the AAV-Cd82 injection group was much higher than that in the vehicle and control groups, covering the retina and ONH and particularly pronounced in the optic nerve (Fig. 2a). The western blot results also revealed satisfactory transfection efficiency (Fig. 2b, c). Immunofluorescence of the virus tag mCherry colabeled with Tuj1 is presented in Fig. S1.

### CD82 overexpression protected against axonal transport deficits after AOHT

Here, we focused on lesions in the ONH, which is a critical site in glaucoma optic neuropathy [33], and the superior colliculus (SC), which is the primary projection site of RGCs in the rodent brain [3].

**Fig. 3:**
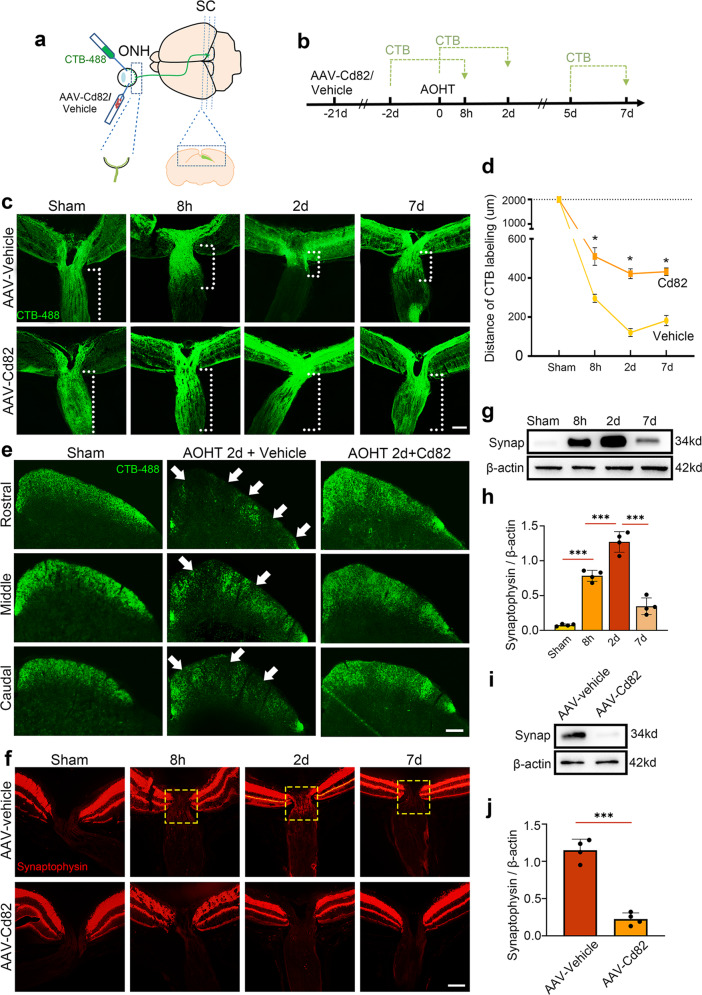
CD82 overexpression protected against axonal transport deficits after acute ocular hypertension. **a** Schematic drawing of AAV injection and CTB tracing from retina to SC. The dashed box indicating the observation site of ONH and SC. **b** Timeline of the major procedures for studying optic nerve transport. **c** Representative images showing anterogradely transported CTB-488 in ONH and proximal ON in groups of Sham, 8 h, 2d and 7d post-AOHT with intravitreal injection of AAV-vehicle or AA-Cd82 obtained at low exposure time of fluorescence microscopy. The white dashed lines indicating the distance of CTB labeling. Scale bar, 50 μm. **d** Statistical analysis of the CTB labeling distance indicated in (**c**). The data were expressed as mean ± S.E.M., *n* = 5 eyes, Student’s t-test for Cd82 v.s. vehicle at each time point, *p < 0.05. **e** Representative images showing CTB-488 transported to SC from three layers as Rostral, Middle, and Caudal in groups of Sham and 2d post-AOHT with intravitreal injection of AAV-vehicle or AA-Cd82 obtained at high exposure time of fluorescence microscopy. The white arrow indicating defects of CTB distribution, *n* = 5 mice. Scale bar, 200 μm. **f** Immunostaining of Synaptophysin within ONH in the same groups as in (**c**). The yellow dashed box indicating accumulation of synaptophysin protein in ONH, *n* = 5 eyes. Scale bar, 50 μm. **g** Western blot showing protein levels of Synaptophysin in ONH tissues in groups of Sham, 8 h, 2d, and 7d post-AOHT. **h** Statistical analysis of the data shown in (**g**), *n* = 4 eyes, one-way ANOVA, ***P < 0.001. **i** Western blot showing protein levels of Synaptophysin in ONH tissues in groups of 2d-post AOHT with injection of AAV-vehicle or AA-Cd82. **j** Statistical analysis of the data shown in (**i**). The data were expressed as mean ± S.E.M., *n* = 4 eyes, one-way ANOVA, ***P < 0.001.

First, we visualized the anterograde transport function of the optic nerve by an intraocular injection of cholera toxin β-subunit (CTB), which labeled the entire retinal projection via active uptake and transport (Fig. 3a, b). Sections of ONH showed that CTB was uniformly distributed along the entire axon length (>2000 μm) in the sham group. Furthermore, after inducing AOHT, CTB failed to be transported distantly, with a severe obstacle on Day 2 (<200 μm) and slight recovery on Day 7 (Fig. 3c, d). Notably, the CD82 overexpression reversed this obstruction and maintained the CTB labeling distance at over 400 μm on Day 2.

The CTB signal density in SC was observed in three layers rostral, middle, and caudal from consecutive serial sections and showed disruption of transport reaching the brain. In the control group, SC had a complete CTB distribution, reflecting entire retinotopic transportation to SC (Fig. 3e). After AOHT, there was a heterogeneous distribution of CTB signals with massive deficits in all three layers. In contrast, SCs from AOHT mice injected with AAV-Cd82 manifested a lower loss of CTB labeling.

Subsequently, we evaluated endogenous cargo transport by immunofluorescence staining of synaptophysin, a vesicular transport marker synthesized by RGCs and anterogradely transported along the ON. Under normal conditions, there was no protein accumulation in the ONH. However, synaptophysin protein began to deposit at the ONH within 8 h after the AOHT modeling and peaked on Day 2, which is similar to the foregoing cases. In the AAV-Cd82 injection group, such accumulation was alleviated (Fig. 3f). Proteins extracted from the ONH were used for a western blot analysis. An increase in synaptophysin protein in the ONH was evident after the modeling, and the protective effect of CD82 was verified (Fig. 3g−j).

### CD82 overexpression protected the optic nerve from axonal degeneration induced by AOHT

Optic nerve axonal degeneration was the following pathological event due to axonal transport deficits and was more severe and difficult to reverse. Here, we detected axonal degeneration changes in the optic nerve after AOHT.

**Fig. 4:**
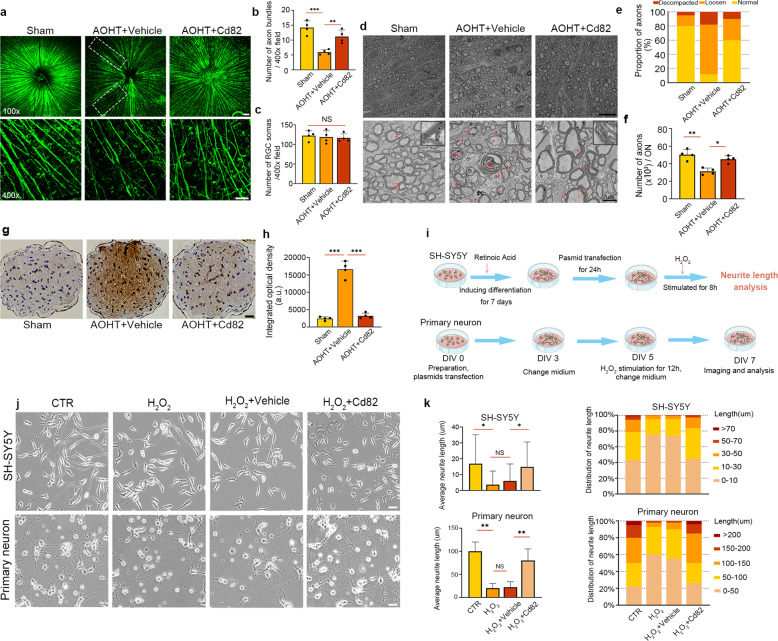
CD82 overexpression protected optic nerve from axonal degeneration induced by AOHT. **a** Immunostaining of Tuj1 in retina flat mount to show RGC axons in groups of Sham and 2d post-AOHT with or without CD82 overexpression. Top panel for images of 100×, scale bar, 100 μm; Bottom panel for images of 400×, scale bar, 50 μm. The white dashed box indicating sparse axon distribution. Statistical analysis of Tuj1^+^ axon bundles (**b**) and Tuj1^+^ RGCs (**c**) shown in the bottom panel of (**a**). The data were expressed as mean ± S.E.M., five nonoverlapping images were chosen for statistical analysis of each retina, *n* = 4 retinas. **d** Electron microscopy images from optic nerve cross-section showing axon damage in groups of CTR and 2d post-AOHT with or without CD82 overexpression. Top panel, scale bar, 10 μm; Bottom panel, scale bar, 2 μm. Red arrowheads denoting delaminated myeline. Red asterisks representing spiral degeneration of axons. The right upper corner in each picture showing the magnification of myelin sheath in red dashed boxes. Statistical analysis of axon proportions with normal, loosen, or decompacted myelin (**e**) and the number of myelinated axons per ON (**f**) as shown in (**d**). Five nonoverlapping images were chosen for statistical analysis of each individual, *n* = 4 optic nerves. **g** Immunohistochemical staining of Aβ in optic nerve cross-section grouped as in (**a**). Scale bar, 20 μm. **h** Statistical analysis of integrated optical density shown in (**g**), *n* = 4 optic nerves. **i** Schematic drawing of the major procedures in neurite outgrowth analysis for SH-SY5Y cells and primary hippocampal neurons. **j** Representative images showing neurite outgrowth of SH-SY5Y cells and primary hippocampal neurons in control group and H_2_O_2_ groups with or without Cd82 plasmid transfection. Scale bar, 20 μm. **k** Statistical analysis of neurite length shown in (**i**). Twenty nonoverlapping fields of view chosen for analysis in each group for one experiment, *n* = 200 cells. Statistical tests in (**b**), (**c**), (**f**), (**h**) and (**k**) using one-way ANOVA, *P < 0.05, **P < 0.01, ***P < 0.001.

Radial cord-like immunofluorescence staining of Tuj1 in the nerve fiber layer demonstrated the axons of ganglion cells. Axon loss occurred in AOHT group, which showed weakened immunofluorescence and sparser axon distribution. With CD82 overexpression, the axon distribution was nearly intact (Fig. 4a, b). However, there was no significant loss of RGC soma at this stage, indicating that axonal degeneration is an early pathological change prior to RGC death (Fig. 4c). The existing time lag between earlier axon loss and later RGC body death is consistent with previous findings [34].

Electron microscopy showed the ultrastructural pathological changes of RGC axonal degeneration [35, 36]. Representative images of control, AOHT, and CD82 intervention groups are shown in Fig. 4d. In the normal group, compact layers of myelin lamellae surrounded the axons. However, two days after AOHT, myelinated axons appeared to be delaminated with intramyelinic lacunae or vacuoles. Myelin debris and axonal swellings were frequently observed, and the emergence of almost collapsed axon structures with multilayered whorled masses indicated axons toward the end stage of degeneration. To quantify the degree of damage, the proportion of axons with normal, loosened, or decompacted myelin was calculated (Fig. 4e). In the AOHT group, most myelin sheath was loosened, with another considerable fraction decompacted. With CD82 overexpression, this impairment was alleviated and close to normal. Myelinated axon counts also reflected the protective role of CD82 in AOHT model (Fig. 4f).

Extensive studies have revealed that β-amyloid precursor protein (Aβ) is involved in glaucomatous neuropathology [37], and Aβ has emerged as a hallmark of neurodegeneration [38, 39]. As shown in Fig. 4g, h, axons immunoreactive to Aβ were present to a significantly lower degree in the CD82-overexpressing mice than the vehicle-treated group after modeling.

To further verify the protective effect of CD82 overexpression in vitro, we used retinoic acid (RA)-differentiated SH-SY5Y cells [40] as well as mouse primary hippocampal neuron for neurite outgrowth assays. H_2_O_2_ (100 μM) was applied to imitate cell injury in retinal degeneration [41]. As shown in Fig. 4i−k, H_2_O_2_ led to cell process retraction and inhibited the neurite length of both cells. With CD82 overexpression, axonal outgrowth was almost restored to the normal state after H_2_O_2_ injury.

### CD82 overexpression promoted axon regeneration, RGC survival, and visual function in an optic nerve crush (ONC) model

**Fig. 5:**
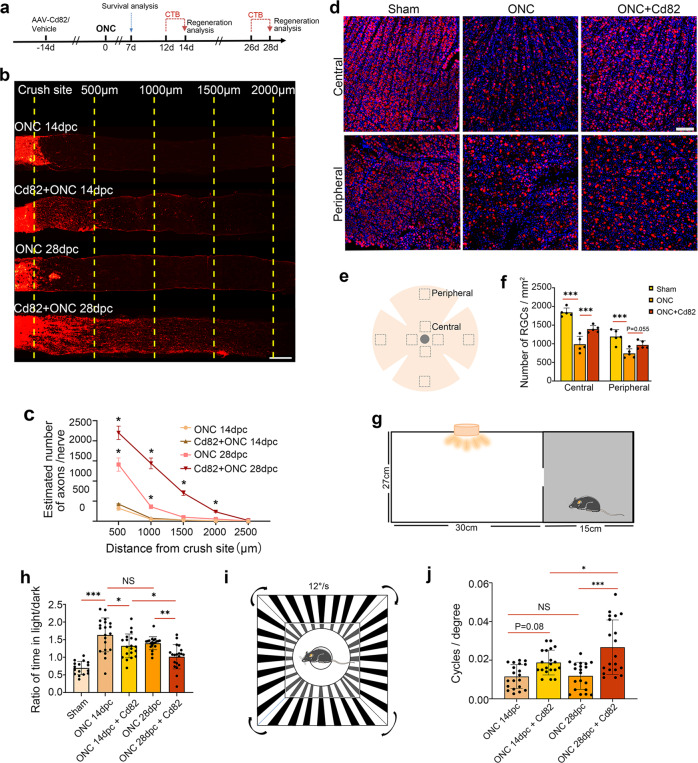
CD82 overexpression promoted axon regeneration, RGC survival, and visual function after optic nerve crush. **a** Timeline of the major procedures for exploring optic nerve regeneration and RGC survival after ONC. **b** Representative images showing axonal regeneration of optic nerve 14 or 28 days after ONC with or without CD82 overexpression. The yellow dashed line indicating the distances from the crush site. Scale bar, 200 μm. (dpc = day post crush). **c** Statistical analysis of regenerated axons shown in (**b**), *n* = 4 optic nerves, one-way ANOVA, *p < 0.05 for CD82 + ONC 14dpc v.s ONC 14dpc and CD82 + ONC 28dpc v.s ONC 28dpc. **d** Immunostaining of Rbpms in retina flat mount to mark RGC somas in control group and 7 day-post-ONC groups with or without CD82 overexpression. Scale bar, 100 μm. **e** Schematic indicating observation area in retina flat mount. **f** Quantification of RGC numbers shown in (**d**). Four nonoverlapping fields of view chosen for analysis in each retina, *n* = 4 retinas, one-way ANOVA, ***P < 0.001. **g** Schematic drawing of mouse dark/light preference tests to evaluate light perception. **h** Statistical analysis of mouse dark/light preference tests. **i** Schematic drawing of mouse optomotor response tests to evaluate visual acuity. **j** Statistical analysis of mouse optomotor response. Statistical tests in (**h**) and (**j**) using one-way ANOVA, *n* = 20 mice, *P < 0.05, **P < 0.01, ***P < 0.001.

Currently, there are limited means to reverse optic nerve damage and restore eyesight in late-stage glaucoma with extensive axonal injury and RGC loss [42]. Here, we induced CD82 overexpression in a more dramatic injury model, namely, optic nerve crush (ONC), which led to evident neuronal loss and rapid axonal damage by directly interrupting axoplasmic transport [43] comparable to advanced glaucoma (Fig. 5a).

Figure 5b presents typical images of CTB-traced axon regeneration after ONC. Regenerating and sprouting RGC axon fibers extended over 1 mm beyond the crush site on Day 14 and extended to 2 mm on Day 28 under the condition of CD82 overexpression, with no visible regeneration in the injury-only group regardless of the recovery time (Fig. 5c). In addition, RGC survival was evaluated by RBPMS immunofluorescence staining in retinal flat mounts 7 days after ONC (Fig. 5d). The promotion of RGC survival following the CD82 overexpression was significant in the central retina, while the effect in the peripheral area was not as evident (Fig. 5e, f).

Then, we tested the function of RGCs through mouse visual function tests. Dark/light preference tests were performed to measure the light perception (LP) of mice with bilateral ONC; during the test, a reduced duration in the dark compartment reflected a loss of vision (Fig. 5g). The tests revealed that the ratio of time in the light/dark was increased after ONC and significantly recovered in the CD82 overexpression group at both time points, indicating a restoration of LP after the treatment with CD82 (Fig. 5h). High-contrast visual stimulation was performed to measure the optomotor response to reflect the visual acuity of the mice (Fig. 5i). The high spatial frequency threshold indicated improved visual acuity in the AAV-Cd82-injected mice compared to that in the injury-only group on Day 28 after bilateral ONC (Fig. 5j). Overall, the restored vision-dependent behavior of the mice following CD82 overexpression demonstrated its ability to improve visual function after optic nerve injury.

### CD82 overexpression protected against axonal transport deficits through an mTORC1-dependent mechanism

**Fig. 6:**
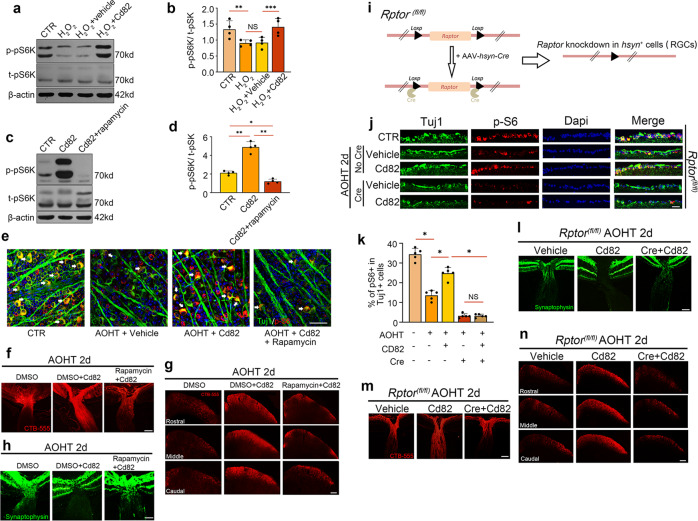
CD82 overexpression protected against axonal transport deficits through a mTORC1-dependent mechanism. **a** Western blot analysis of phosphorylated-pS6K and total pS6K in HEK-293T cells in CTR group and H_2_O_2_ groups with or without Cd82 plasmid transfection. **b** Statistical analysis of the data shown in (**a**), *n* = 4. **c** Western blot analysis of phosphorylated pS6K and total pS6K in HEK-293T cells in CTR group and Cd82 plasmid transfected groups with or without Rapamycin. **d** Statistical analysis of the data shown in (**c**), *n* = 4. **e** Co-immunofluorescent staining of p-S6 (red) with RGC marker Tuj1 (green) in retina flat mount. Scale bar, 50 μm. **f** Representative images showing anterogradely transported CTB-555 in ONH and proximal ON in Cd82 overexpression groups with or without rapamycin 2d after AOHT, *n* = 5 eyes. Scale bar, 100 μm. **g** Representative images showing CTB-555 transported to SC from three layers as Rostral, Middle, and Caudal, *n* = 5 mice. Scale bar, 200 μm. **h** Immunostaining of Synaptophysin within ONH, *n* = 5 eyes. Scale bar, 100 μm. **i** Schematic drawing of Cre induced conditional knockdown of Raptor in RGCs in *Rptor*^*fl/fl*^ mouse. **j** Co-immunofluorescent staining of p-S6 (red) with RGC marker Tuj1 (green) in ganglion cell layer (GCL) of retina sections in vehicle or Cd82 overexpression groups with or without knockdown of Raptor by Cre. Scale bar, 50 μm. **k** Statistical analysis of p-S6 and Tuj1 co-labeling in GCL shown in (**j**), *n* = 5 eyes. **l** Immunostaining of Synaptophysin within ONH in Cd82 overexpression groups with or without knockdown of Raptor 2d after AOHT, *n* = 5 eyes. Scale bar, 100 μm. **m** Representative images showing anterogradely transported CTB-555 in ONH and proximal ON, *n* = 5 eyes. Scale bar, 100 μm. **n** Representative images showing CTB-555 transported to SC from three layers as Rostral, Middle, and Caudal, *n* = 5 mice. Scale bar, 200 μm. Statistical tests in (**b**), (**d**) and (**k**) using one-way ANOVA, *P < 0.05, **P < 0.01, ***P < 0.001.

The mTOR signaling pathway is critical for regulating neuronal function. Subsequently, we speculated whether the mTOR pathway is required for the CD82-mediated protective effects. The western blot analysis shown in Fig. 6a, b illustrated that phosphorylated-pS6K was downregulated in the H_2_O_2_-treated cells but recovered following CD82 overexpression. Furthermore, the CD82-induced increase in phosphorylated pS6K was blocked by rapamycin, a potent and specific inhibitor of mTOR, confirming the participation of the mTORC1 pathway downstream of CD82 (Fig. 6c, d). These regulatory relations could also be corroborated in retinal tissues as shown in Fig. 6e. The double immunofluorescence staining in retinal flat mounts of phosphorylated S6 and the RGC marker Tuj1 was decreased in the AOHT group, while the CD82 overexpression prevented this decline. The treatment with rapamycin diminished the signal activation effect of CD82.

To further validate the role of mTORC1 activity in regulating the axonal transport function promoted by CD82, we repeated the previous experiments with the addition of rapamycin treatment every two days for 28 days to block mTOR activity in vivo. In this case, the overexpression of CD82 failed to relieve the axonal transport deficits induced by AOHT, which manifested in the accumulation of synaptophysin protein at the ONH (Fig. 6h) and impairment in CTB transport from the eye to SC (Fig. 6f, g). Considering the confounding effect of the pharmacological inhibitor rapamycin due to systemic drug administration and low tissue specificity, we used conditional knockout mice to target RGCs by injecting AAV*-hsyn-cre* into the vitreous body of *Rptor*^*fl/fl*^ mice to delete Raptor, the exclusive functional component of mTORC1, to verify the specific effect of the mTORC1 pathway in RGCs (Fig. 6i). The results of the species identification and the Raptor knockdown efficiency in the retina are shown in Fig. S2. Figure 6j, k illustrates the deactivation of the mTORC1 pathway in the Cre-injected eyes from the *Rptor*^*fl/fl*^ mice. The assessment of axonal transport shown in Fig. 6l−n was consistent with the previous results showing that the inhibition of mTORC1 activity in RGCs blocked the protective effects of CD82.

### CD82 activated mTORC1 via TRAF2-mediated k63-linked ubiquitylation to protect against neurodegeneration

**Fig. 7:**
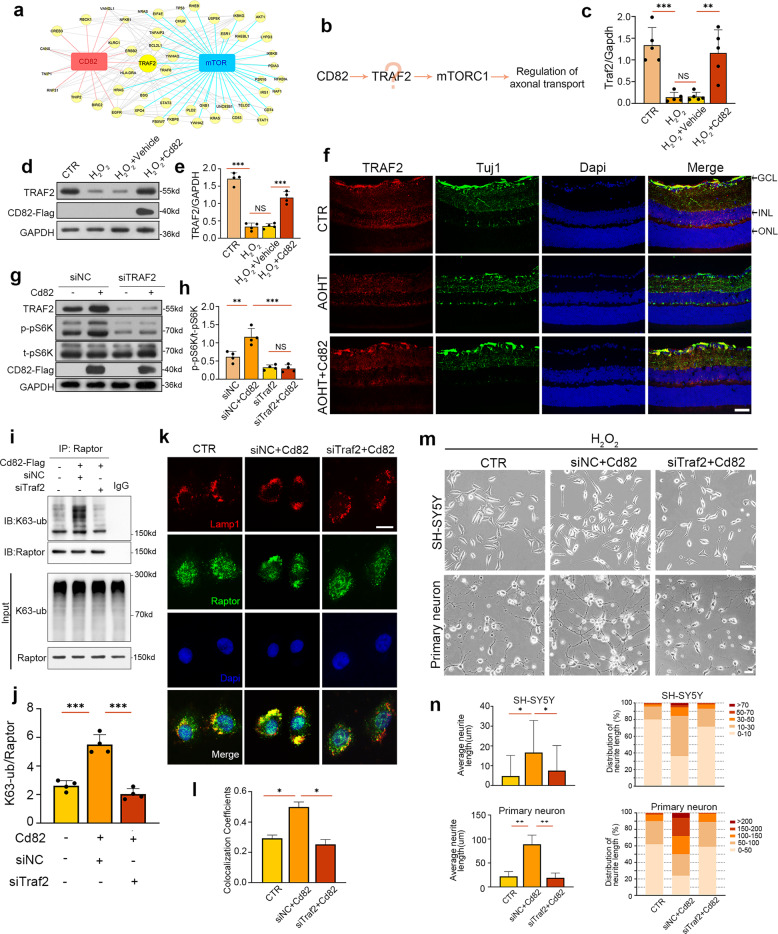
CD82 activated mTORC1 via TRAF2 mediated k63-linked ubiquitylation to protect against neurodegeneration. **a** PPI network of hub molecules connecting CD82 with mTOR. Node size reflected the compositive correlation with CD82 and mTOR. **b** The putative pathway of TRAF2 mediated activation of mTORC1 downstream of CD82. **c** RT-PCR analysis of Traf2 mRNA in HEK-293T cells in CTR group and H_2_O_2_ groups with or without Cd82 plasmid transfection, *n* = 5. **d** Western blot analysis of TRAF2 protein level in HEK-293T cells in CTR group and H2O2 groups with or without Cd82 plasmid transfection. **e** Statistical analysis of the data shown in (**d**), *n* = 4. **f** Immunostaining of TRAF2 (red) with RGC marker Tuj1 (green) in retina sections of control group and 2d post-AOHT groups with or without AAV-Cd82, *n* = 5 retinas. Scale bar, 50 μm. GCL = ganglion cell layer, INL = inner nuclear layer, ONL = outer nuclear layer. **g** Western blot analysis of phosphorylated pS6K and total pS6K in HEK-293T cells in siNC group or siTraf2 group with or without Cd82 plasmid transfection. **h** Statistical analysis of the data shown in (**g**), *n* = 4. **i** Immunoprecipitation of K63-linked ubiquitination with raptor in control group and Cd82 overexpression groups with or without siTraf2. **j** Statistical analysis of the data shown in (**i**), *n* = 4. **k** Co-immunofluorescent staining of Raptor (green) with the lysosome marker Lamp1 (red) in SH-SY5Y cells of control group and Cd82 overexpression groups with or without siTraf2. Scale bar, 20 μm. **l** Statistical analysis of Raptor and Lamp1 colocalization in SH-SY5Y cells shown in (**k**), *n* = 50 cells. **m** Representative images showing neurite outgrowth of SH-SY5Y cells and primary hippocampal neurons in the same group as in (**k**). Scale bar, 20 μm. **n** Statistical analysis of neurite length shown in (**m**), *n* = 200 cells. Statistical tests in (**c**), (**e**), (**h**), (**j**), (**l**) and (**n**) using one-way ANOVA, *P < 0.05, **P < 0.01, ***P < 0.001.

Subsequently, the molecular pathway by which CD82 influences mTORC1 activation was addressed. We utilized the Molecular Interaction Search Tool (MIST) database to extract hub molecules connected to CD82 and mTORC1 from the protein–protein interaction (PPI) network. The consequent protein interaction map was visualized by Cytoscape software. The node size reflected the compositive correlation between the candidate proteins and CD82 and mTOR. As demonstrated in Fig. 7a, tumor necrosis factor receptor-associated factor 2 (TRAF2) was the most likely intermediate molecule due to its direct association with both target proteins (Fig. 7b). Then, we examined the mRNA level of Traf2 by RT–PCR (Fig. 7c). Further western blot analysis was consistent with the PCR results, indicating that CD82 overexpression caused the upregulation of TRAF2 (Fig. 7d, e). To verify our ex vivo findings in vivo, we evaluated the expression of TRAF2 in the mouse retina. Figure 7f shows that the expression of TRAF2 in Tuj1-positive GCL was markedly decreased, while the CD82 overexpression suppressed this decrease. The results above indicate that TRAF2 is a downstream effector regulated by CD82.

The impact of the CD82-induced upregulation of TRAF2 on mTORC1 activation was explored by a western blot analysis of phosphorylated pS6K. The CD82 overexpression led to increased protein levels of TRAF2, which, in turn, activated the mTORC1 pathway (Fig. 7g, h). To validate whether the CD82-induced activation effect was dependent on TRAF2, we used siRNA to knockdown Traf2 in Cd82-overexpressing cells. In this case, CD82 was no longer capable of activating the downstream phosphorylation of pS6K (Fig. 7g, h), indicating a TRAF2-dependent mechanism underlying the CD82-induced effect.

It has been demonstrated that K63-linked ubiquitination regulates the activity of mTORC1 [44, 45]. Therefore, we speculated that TRAF2-mediated K63-linked ubiquitination participates in mTORC1 activation. As shown in Fig. 7i, j, a high level of K63-linked ubiquitination was observed in the CD82-overexpressing cells, which was attenuated by siTraf2 transfection. Therefore, we are confident that CD82 acted by upregulating TRAF2, which was capable of ubiquitinating RAPTOR to govern mTORC1 activation. Further coimmunofluorescence analysis demonstrated increased colocalization of RAPTOR with the lysosomal marker LAMP-1 in the CD82-overexpressing SH-SY5Y cells, which was reduced in the siTraf2-transfected cells (Fig. 7k, l).

Finally, we tested the effect of TRAF2 on neurodegenerative pathology with a neurite outgrowth assay. Figure 7m, n shows that the CD82-induced outgrowth-promoting capability was retarded by siTraf2, confirming that CD82 protected against neurodegeneration through the positive regulation of TRAF2.

## Discussion

**Fig. 8:**
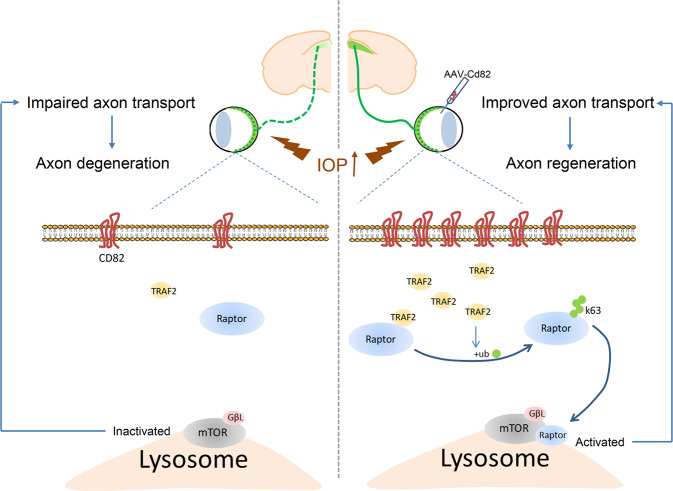
Schematic representation of CD82 neuroprotection effect after ocular hypertension. Acute ocular hypertension induced axonal transport deficits and subsequently caused axon degeneration. CD82 overexpression protected against axon injury through upregulating TRAF2, which activates mTORC1 through K63-linked ubiquitylation and intracellular repositioning of Raptor.

Altogether, our study revealed the protective effect of CD82 against axonal transport deficits and subsequent axon degeneration after AOHT via mTORC1 activation (Fig. 8).

Our study demonstrates the spatial characteristics and timeframes of the key early pathological events in an acute glaucoma model. Axonal transport impairment initiated at the ONH and progressed to involving the brain (i.e., superior colliculus), which occurred as early as 8 h after IOP elevation, reached the highest level on Day 2 and then gradually declined. Such spontaneous recovery was insufficient to successfully reverse the subsequent axonal degeneration, at which point the RGC soma remained intact. The pathological progression in this study was consistent with previous research using other glaucoma models [46–48]. A more recent study using Dex-induced ocular hypertension demonstrated axonal transport deficits and axonal degeneration preceding RGC structural and functional loss. The partial blockade of axonal transport was associated with the early stages of optic nerve degeneration [49]. However, the axonal transport deficits in such research were progressively aggravated due to sustained IOP elevation, making it difficult to inquire about and intervene with the early reversible alterations. By inducing AOHT at a stable level of 75 mmHg for 1 h, we found that the transport deficits were still reversible under this condition, while axonal degeneration was already initiated. Hence, early interventions targeting axonal transport function are more meaningful for protecting against irreversible end-stage neuropathy in glaucoma.

Here, we identified the protective effect of CD82 in promoting axonal transport and subsequently protecting against neurodegeneration in an AOHT model. Notably, the variation trend of the CD82 protein levels was consistent with the severity of axonal transport disorders after AOHT. Therefore, the reduced CD82 levels corresponded to aggravated axonal dysfunction with time synchronization after injury. The spatial localizations of CD82 mainly in RGCs and their axons further indicated its direct role in regulating RGC axonal functions. Thus, we applied AAV-mediated Cd82 overexpression specifically in RGCs as a complementary gene therapy. In addition, we illustrated the pro-regenerative effects of CD82 in a more severe injury model of ONC in which axon loss and RGC death were apparent due to complete axoplasmic flow disruption comparable to advanced glaucoma. Upon AAV-Cd82 injection, axon regeneration and vision restoration were achieved 28 days after injury. This extent of recovery was approximately the same as that reported following other pro-regenerative strategies. To date, most approaches aimed to improve different neuron intrinsic capacities, such as targeting key signaling pathways [50, 51], manipulating transcription factors [40], and regulating epigenetic mechanisms [52]. Furthermore, promoted axonal transport has been proven to increase axonal regeneration in many studies [53–55]. Here, we confirmed the pro-regeneration effect of CD82 in a mouse ONC model, which could be partially due to the enhancement of axonal transport demonstrated above.

Our data show that CD82 overexpression reverted the decrease in p-pS6k induced by injury, indicating that the activation of mTORC1 underlies the protective effect. The mTOR pathway has been reported to function downstream of various signaling molecules to promote axonal regeneration [56–59]. In addition, a growing number of researches have revealed the important role of autophagy regulated by mTORC1 in glaucomatous optic neuropathy [60]. However, controversial results arose due to varying models, complex cell types, as well as different time course, studied [61, 62]. Most evidence supports the protective effect of rapamycin as an autophagy inducer in alleviating RGC loss and optic nerve degeneration in glaucoma models [63, 64]. On the contrary, some studies found inhibition of autophagy to be neuroprotective [65, 66]. mTORC1 functions as a suppressor of autophagy, taking variable effect depending on a different context. Our study demonstrated mTORC1 activation mediated by CD82 to be protective in glaucomatous neurodegeneration, the involvement of autophagic pathway in this process remains to be further explored.

The PPI data obtained from the MIST database suggest that TRAF2 plays mediating role in the interaction between CD82 and the mTORC1 pathway. A recent study indicated that K63-linked polyubiquitination was critical for mTORC1 activation and proved that TRAF6 mediated mTORC1 translocation to the lysosome for subsequent activation [30]. Another study investigating TRAF2 further emphasized its role in the ubiquitination of mTOR components to maintain the balanced homeostasis of mTORC1 and mTORC2 activation [67]. Here, we demonstrated a novel mechanism by which TRAF2 functions downstream of CD82 to activate mTORC1 by ubiquitinating Raptor. However, the mechanisms by which CD82 upregulates TRAF2 remain to be elucidated.

Current research investigating the tetraspanin CD82 mainly focuses on tumor metastasis regulation and immune recognition [68, 69]. Research concerning tetraspanin function in the nervous system is still at an early stage; however, some evidence indicating the roles of other tetraspanins in the regulation of the nervous system has gradually emerged [70–73]. To the best of our knowledge, our study is the first to explore the role of CD82 in neurodegenerative disease in eyes, which helps fill this gap, gain deeper insight into tetraspanins, and provide a potential neuroprotective strategy for optic nerve protection.

## Materials and methods

### Animals

Male C57BL/6J mice and B6. Cg-*Rptor*^*tm1.1Dmsa*/J^ (The Jackson Laboratory, 013188) were housed in the Animal Center of the Tongji Medical College. All experimental animals were given free access to food and water in a 12 h light/12 h dark cycle environment. All animals were treated under protocols approved by ARVO Statement for the Use of Animals in Vision and Ophthalmic Research and the institutional IACUC committees of Huazhong University of Science and Technology. Animals of 2-month-old weighing 20−25 g were used in all experiments.

### Reagents and antibodies

Retinoic acid was obtained from Sigma-Aldrich China (Shanghai, China; R2625). Rapamycin was purchased from MedChemExpress (Shanghai, China; HY-10219). CTB-Alexa 488 and CTB-Alexa 555 were bought from BrainVTA (Wuhan, Hubei, China). The following antibodies were used in western blot: anti-CD82 (ab66400 & ab135779; Abcam; 1:1000); anti-synaptophysin (ab14692; Abcam; 1:1000); anti-p70S6k (2708T; Cell Signaling Technology; 1:1000); anti-p-p70S6k(9234T;Cell Signaling Technology; 1:1000); anti-β-actin (sc-47778; Santa Cruz; 1:1000); anti-TRAF2 (ab126758; Abcam; 1:1000); anti-Raptor (20984-1-AP; Proteintech; 1:1000).The following antibodies were used in immunofluorescence: anti-CD82 (ab66400; Abcam; 1:200); anti-Tuj1 (801201; Biolegend; 1:1000); anti-Iba1(ab48004;Abcam;1:100); anti-GAFP(ab4674; Abcam;1:100); anti-mCherry (ab167453; Abcam; 1:100); anti-synaptophysin (ab14692; Abcam; 1:200); anti-pS6(Ser 240/244)(5364T; Cell Signaling Technology; 1:1000); anti-TRAF2 (ab126758; Abcam; 1:400); anti-Raptor (20984-1-AP; Proteintech; 1:1000); anti-Lamp1 (15665, Cell Signaling Technology; 1:500).

Anti-β-Amyloid (2450; Cell Signaling Technology; 1:100) was used in immunohistochemistry.

### Adeno-associated virus administration

rAAV2/9-hsyn-Cd82-2A-mCherry-WPRE-PA, rAAV2/9-hsyn-mCherry-WPRE-PA, rAAV2/9-hsyn-Cre-EGFP-WPRE-PA, rAAV2/9-hsyn-EGFP-WPRE-PA were obtained from BrainVTA (Wuhan, Hubei, China). Each virus preparation contained approximately 2.0 × 10^12^ genome copies/ml. One microliter of AAV was injected in one eye of each animal in the vitreous cavity using a 35-G needle with a 10 μl Hamilton microsyringe (Hamilton, Reno, NV, USA) at a constant rate over 30 s. The needle was held in place for 60 s to allow for intraocular pressure equilibration before removal. Animals were used for subsequent experiments three weeks after AAV injection.

### Animal model of Acute Ocular Hypertension

Mouse model of acute glaucoma was performed as previously described [74]. Animals undergoing surgery were anesthetized by intraperitoneal injections of 5% chloral hydrate (9 ml/kg). The corneas were topically anesthetized with 0.5% tetracaine hydrochloride, and the pupils were dilated with 1% tropicamide. A 30-gauge infusion needle connected to a standard saline reservoir was used to insert into the anterior chamber of 1 eye. The saline reservoir was elevated to a height of 1.2 m for 1 h. Whitening of the iris and loss of the red reflex suggested the retinal ischemia, and subsequent reperfusion was evident by the red reflex return. The other eye was served as a control with a sham procedure performed without elevating the pressure. After the process, eyes were covered with antibiotic ointment to prevent corneal desiccation and bacterial infection. The animals were allowed to recover for 8, 24, 48, 72 h or 7 days before sacrifice.

### Optic nerve crush

Mice were anesthetized by intraperitoneal injections of 5% chloral hydrate (9 ml/kg), and eyes were locally anesthetized using 0.5% tetracaine hydrochloride. A small conjunctival incision was made in the superior posterior area using micro-scissors, and eye muscles were then carefully moved. The optic nerve was exposed intraorbitally and crushed by fine self-closing forceps for 5 s approximately 0.5 mm behind the optic disc without damaging the underlying ophthalmic artery. Eyes were covered with antibiotic ointment to protect the cornea after surgery. Mice were euthanized at day 7 post-injury for RGC survival analysis and day 14 or day 28 for axonal regeneration evaluation.

### Immunofluorescence

Eyes for retina cross-sectional preparation were fixed in 4% paraformaldehyde (pH = 7.4) as whole globes at RT for 2 days. All specimens were embedded in paraffin to enable ONH to be cut in parallel with ON longitudinal axis. The paraffin-embedded retinal sections (5 μm) were gently washed three times with phosphate-buffered saline (PBS) pre-heated to 37 °C and then blocked in 5% donkey serum albumin for 1 h to avoid nonspecific binding. Afterward, the sections were incubated in diluted primary antibodies at 4 °C overnight. Retinal paraffin sections were washed three times before incubated with the conjugated secondary antibody in 5% bovine serum albumin (RT, 90 min).

Eyes for retinal flat mount were dissected and fixed in 4% paraformaldehyde (pH = 7.4) for 2 h at room temperature. The posterior segments of the eye were cut into a ‘petal’ shape with 4 to 5 radial incisions, and then the retinas could be carefully detached. Leave the retinas to cold methanol for at least 20 min to facilitate permeabilization. After rinsed in PBS, retinas were blocked in normal donkey serum for 1 h at room temperature and then incubated for 48−72 h at 4 °C with primary antibodies. Afterward, retinas were washed thoroughly in PBS three times and incubated with secondary antibodies at room temperature for 90 min. Finally, retinas were transferred onto slides and mounted with glycerol.

The sections and flat mounts were examined with a laser scanning confocal microscope (Zeiss LSM 710; Zeiss, Oberkochen, Germany) under excitation wavelengths of 405 nm for DAPI, 488 nm for FITC, and 594 nm for cy3, respectively.

For colocalization analysis, Mander’s colocalization coefficient (MCC) was calculated using the ImageJ plugin’JACoP’.

### Evaluation of anterograde axon transport by CTB

Mice were anesthetized by 5% chloral hydrate and mydriasis with 1% tropicamide.1 μl CTB-Alexa 488 (BrainVTA, Wuhan, Hubei, China) was intravitreally injected in one eye using a 10 μl Hamilton microsyringe (Hamilton, Reno, NV, USA). Forty-eight hours after the injection, animals were anesthetized and sacrificed via cardiac perfusion of normal saline and 4% PFA. Brains and eyes were post-fixed in 4% PFA for an additional 24 h, dehydrated with 30% sucrose in PBS overnight prior to embedding in OCT (Tissue-Tek, Sakura Finetek Inc, Tokyo, Japan). Brains were continuously sliced into 30 μm sections in area of superior colliculus. Eyes were sectioned into 10 μm with ONH. Alexa 488 was visualized using a fluorescent microscope (Olympus, Tokyo, Japan).

### Axon labeling for regeneration

CTB-Alexa 555 (BrainVTA, Wuhan, Hubei, China) was intravitreally injected 48 h before sacrifice to trace regenerating RGC axons 14- or 28-days post-injury. After 4% PFA perfusion, mice eyes and optic nerves were microdissected and post-fixed for 3 h in 4% PFA. The nerves were dehydrated with 30% sucrose in PBS overnight at 4 °C and embedded in OCT Compound (Tissue-Tek, Sakura Finetek Inc, Tokyo, Japan) for cryosection. Optic nerves were cut longitudinally at a thickness of 10 µm and mounted onto slides. Alexa 555 was imaged using a fluorescent microscope (Olympus, Tokyo, Japan). Regeneration axons were quantified by counting the number of CTB-labeled fibers extended past every 500 µm division from the crush site. The total number of regeneration axons in each optic nerve was estimated using the equation elaborated in the literature [56].

### Electron microscopy and analysis

Eyes with optic nerves attached were carefully dissected from the orbit. A 1.5 mm section of optic nerve proximal to the globe was isolated and fixed in ice-cold 2.5% glutaraldehyde in 0.1 M cacodylate solution. Tissue embedding and ultrathin sectioning were processed as described [75]. Sections were examined and photographed with HITACHI H-7000FA TEM.

Axons were classified into three categories according to the condition of the myelin sheath, representing the different degrees of axonal degenerative change. Five non-overlapping visual fields of each section were randomly selected, and the frequency of different degenerative axons was calculated. Observers conducting assessments were masked to the experimental conditions of the images.

### Visual function analysis

Both eyes were injected with AAV-Cd82 or vehicle three weeks before binocular optic nerve crush. Visual function tests were performed on day 14 and day 28 post-injury. For the dark light preference test, apparatus consisting of a small dark chamber and a large illuminated chamber (550 lumens) with a door allowed mice to move freely for 10 min. The time spent in each compartment was recorded by a camera, and the time ratio was calculated automatically by SuperMaze Software (XinRuan Information Technology, Shanghai). For the optomotor response test, the visual acuity of mice was measured based on an innate visual-motor reflex using a testing chamber [76] and the software OptoTrack from XinRuan Information Technology (Shanghai, China). Mice moved freely on a platform located in the center of an area surrounded by four screens displaying a moving vertical sinusoidal grating pattern. The spatial frequency started from 0.01 to 0.06 cycles per degree with constant rotation speed (12°/s) and 100% contrast to determine the spatial frequency threshold at which the mice still tracked the moving grid. Different testing frequencies occurred randomly and repeated 10 times within one test to reduce the occasional error. Observers were blinded to the group of mice.

### Cell culture and transfection

HEK-293T cells were purchased from Boster Biologic Technology. SH-SY5Y cells were given as a gift from the Department of Neurobiology, Tongji Medical College, Huazhong University of Science and Technology.

Cells were maintained in high-glucose DMEM (Hyclone Laboratories, Logan, UT, USA) supplemented with 10% FBS (Gibco, CA, USA) and penicillin (100 U/ml)/streptomycin (100 μg/ml) at 37 °C in a humidified atmosphere with air containing 5% CO2. Confluent cell layers were split every three days.

HEK293 and SH-SY5Y cells were transfected with Flag-Cd82 plasmid/vehicle and siTraf2/siNC using Lipofectamine 3000 (Thermo Fisher Scientific) according to the transfection protocol. Cells were cultured for another 48−72 h for further biochemical analyses. Flag-Cd82 plasmid was constructed by cloning the corresponding cDNA into the pcDNA3-Flag vector. TRAF2 siRNA duplexes (5′-CGACAUGAACAUCGCAAGC-3′) with 30 dTdT overhangs were synthesized at Tsingke biological technology.

Primary hippocampal neurons were cultured as previously described [77]. In brief, primary neurons were prepared from C57BL/6 mouse embryos (E18) using a dissection microscope, then washed with D-Hank’s solution under sterile conditions and seeded on plates coated with poly-L-lysine (50 mg/mL) (Sigma-Aldrich) at a density of 1 × 106 cells/cm^2^. The cells were grown in Neurobasal medium (Thermo Fisher) supplemented with 2% B27 (Gibco), 1% glutamine, and 1% penicillin/streptomycin (Thermo Fisher) at 37 °C in a humidified incubator in air containing 5% CO2. The cultures were composed of almost 99% neuronal cells as estimated by immunocytochemical staining with NeuN. Primary neurons were transfected with plasmids using EntransterTM-D4000 transfection reagent (Engreen Biosystem) or transfected with siRNAs using EntransterTM-R4000 transfection reagent (Engreen Biosystem) following manufacturers’ protocols on the day of preparation (DIV 0).

### Neurite outgrowth assay

RA (all-trans-retinoic acid, Sigma) was used to induce cell differentiation and neurite outgrowth as indicated previously [78]. Briefly, SH-SY5Y cells with proper confluence were cultured in 1%FBS medium supplemented with 10 μM RA for 7days prior to treatment. On day 4, the medium was replaced with fresh differentiation medium, and on day 7, cells were used for subsequent experiments.

Oxidative stress induced by hydrogen peroxide exposure was indicated to cause axonal degeneration in vitro [41]. SH-SY5Y cells and primary hippocampal neurons were exposed to 100 μM hydrogen peroxide for 8 h to inhibit neurite outgrowth and recovered in normal medium for another 12 h before morphological analysis.

The formation of neurites was observed using an inverted IX71 microscope system (Olympus, Tokyo, Japan). The neurite length of each cell was measured by Image J software plugin’AxonJ’. The average length of neurites and the ratio of cells with different neurite lengths were analyzed.

### Drug handling and administration

Retinoic acid (R2625, Sigma) powder was prepared in DMSO at 3 mg/ml (0.01 M) as stock solution and stored in light-protected vials at −80 °C. Tissue culture medium was used to dilute the stock solution at a final concentration of 10 µM to induce differentiation of SH-SY5Y cells.

For animal experiments, rapamycin (HY-10219, MedChemExpress) was dissolved in DMSO at 20 mg/ml for temporary storage in −20 °C. Before each administration, rapamycin stock solution was diluted in sterile saline solution and given intraperitoneally at 6 mg/kg every two days. For cell experiments, rapamycin was dissolved in DMSO at 20 mM. Subsequent dilutions were made in growth medium with a final concentration of 100 nM and maintained for 2 h before cells were harvested.

### PPI network visualization

To identify new interaction partners and the corresponding interaction networks between CD82 and mTORC1, we used the online database Molecular Interaction Search Tool (MIST; http://fgrtools.hms.harvard.edu/MIST/) [79] and visualized the protein−protein interaction networks by Cytoscape software 3.8.0 [80].

### Protein extraction, immunoblot, and immunoprecipitation

Cells and tissue from retina, ONH, and ON were lysed in RIPA buffer (Applygen Technologies, Beijing China) respectively at designed time-points. BCA Protein Assay Reagent (Boster Biologic Technology) was used to quantify protein concentration. For immunoprecipitation, the same amounts of whole-cell lysate were incubated with the primary antibodies (0.5−2 μg) overnight at 4 °C. Protein A/G sepharose beads (P2012, Beyotime Biotechnology) were added into the incubation tubes, and the mixture was incubated at 4 °C with gentle shaking for 3 h. The precipitated complexes were washed five times with RIPA buffer and then mixed with loading buffer (Boster Biologic Technology) and boiled for 5 min. For western blot analysis, equivalent amounts of total protein or immunoprecipitate were fractionated by SDS-PAGE and then transferred to PVDF membrane (MilliporeSigma). Membranes were blocked at room temperature using 5% nonfat milk in TBST buffer for 1 h and treated overnight at 4 °C with diluted primary antibodies. The following day membranes were washed three times with TBST before incubated with horseradish peroxidase-coupled secondary antibodies for 60 min at room temperature. Immunoreactive bands were detected with a chemiluminescence substrate kit (ECLPlus; PerkinElmer Inc, Covina, CA, USA) prior to exposure using either film or digital detection equipment (BLT GelView 6000 pro). Target protein expression levels were quantified using ImageJ software normalized to β-actin or GAPDH level.

### RNA isolation and real-time quantitative PCR

Total RNA was extracted with RNAiso plus (Takara Biomedical Technology, Beijing, China). RNA concentration and quality were assessed on NanoDrop 2000 (Thermo Fisher Scientific). The eligible RNA samples were reverse-transcribed with PrimeScript™RT reagent Kit (RR047A; Takara Biomedical Technology) according to the manufacturer’s instructions. The amplified cDNA templates were diluted and used for quantitative PCR with TB GreenPremix Ex Taq (RR420A; Takara Biomedical Technology) on the Applied Biosystems 7300 Real-Time PCR System (Thermo Fisher Scientific). Primer sequences were designed as follows:

Cd82: forward 5′-TGTCCTGCAAACCTCCTCCS-3′, reverse5′-CCATGAGCATAGTGACTGCCC-3′;

Traf2: forward 5′-GCTCATGCTGACCGAATGTC-3′,

reverse 5′- GCCGTCACAAGTTAAGGGGAA-3′;

Gapdh: forward 5′-GGAGTCCACTGGCGTCTTCA-3′,

reverse 5′- GTCATGAGTCCTTCCACGATACC-3′.

All samples were run in triplicate with blank controls. The relative expression of target genes was calculated by the 2^−ΔΔCT^ method with normalization against Gapdh level.

### Statistical analysis

The results are expressed by mean ± S.E.M from at least three independent experiments, specific sample size as indicated in the context. Graphing and statistical analysis were performed in statistical software Prism (v.7.03; GraphPad Software, La Jolla, CA, USA). Differences within the experimental groups were assessed by Student’s t-test or one-way analysis of variance (ANOVA). *P* values were considered significant for *P* < 0.05.

## Supplementary information

aj-checklist

Suplemental Information

## Data Availability

The datasets used and/or analyzed during the current study are available from the corresponding author (Yin Zhao, Email: zhaoyin85@hust.edu.cn) on reasonable request.

## References

[1] Jonas JB, Aung T, Bourne RR, Bron AM, Ritch R, Panda-Jonas S (2017). Glaucoma. Lancet.

[2] Calkins DJ (2012). Critical pathogenic events underlying progression of neurodegeneration in glaucoma. Prog Retin Eye Res.

[3] Crish SD, Sappington RM, Inman DM, Horner PJ, Calkins DJ (2010). Distal axonopathy with structural persistence in glaucomatous neurodegeneration. Proc Natl Acad Sci USA.

[4] Shirendeb UP, Calkins MJ, Manczak M, Anekonda V, Dufour B, McBride JL (2012). Mutant huntingtin’s interaction with mitochondrial protein Drp1 impairs mitochondrial biogenesis and causes defective axonal transport and synaptic degeneration in Huntington’s disease. Hum Mol Genet.

[5] Nicolas A, Kenna KP, Renton AE, Ticozzi N, Faghri F, Chia R (2018). Genome-wide analyses identify KIF5A as a novel ALS gene. Neuron.

[6] Godena VK, Brookes-Hocking N, Moller A, Shaw G, Oswald M, Sancho RM (2014). Increasing microtubule acetylation rescues axonal transport and locomotor deficits caused by LRRK2 Roc-COR domain mutations. Nat Commun.

[7] Adalbert R, Milde S, Durrant C, Ando K, Stygelbout V, Yilmaz Z (2018). Interaction between a MAPT variant causing frontotemporal dementia and mutant APP affects axonal transport. Neurobiol Aging.

[8] Quigley HA, Addicks EM, Green WR, Maumenee AE (1981). Optic nerve damage in human glaucoma. II. The site of injury and susceptibility to damage. Arch Ophthalmol.

[9] Quigley HA, McKinnon SJ, Zack DJ, Pease ME, Kerrigan-Baumrind LA, Kerrigan DF (2000). Retrograde axonal transport of BDNF in retinal ganglion cells is blocked by acute IOP elevation in rats. Invest Ophthalmol Vis Sci.

[10] Boucheix C, Rubinstein E (2001). Tetraspanins. Cell Mol Life Sci.

[11] Hemler ME (2005). Tetraspanin functions and associated microdomains. Nat Rev Mol Cell Biol.

[12] Bassani S, Cingolani LA, Valnegri P, Folci A, Zapata J, Gianfelice A (2012). The X-linked intellectual disability protein TSPAN7 regulates excitatory synapse development and AMPAR trafficking. Neuron.

[13] Usardi A, Iyer K, Sigoillot SM, Dusonchet A, Selimi F (2017). The immunoglobulin-like superfamily member IGSF3 is a developmentally regulated protein that controls neuronal morphogenesis. Dev Neurobiol.

[14] Kohl S, Giddings I, Besch D, Apfelstedt-Sylla E, Zrenner E, Wissinger B (1998). The role of the peripherin/RDS gene in retinal dystrophies. Acta Anat.

[15] Dong JT, Lamb PW, Rinker-Schaeffer CW, Vukanovic J, Ichikawa T, Isaacs JT (1995). KAI1, a metastasis suppressor gene for prostate cancer on human chromosome 11p11.2. Science.

[16] Albert V, Hall MN (2015). mTOR signaling in cellular and organismal energetics. Curr Opin Cell Biol.

[17] Lipton JO, Sahin M (2014). The neurology of mTOR. Neuron.

[18] Harun-Or-Rashid M, Pappenhagen N, Palmer PG, Smith MA, Gevorgyan V, Wilson GN (2018). Structural and functional rescue of chronic metabolically stressed optic nerves through respiration. J Neurosci.

[19] Yang X, Hondur G, Li M, Cai J, Klein JB, Kuehn MH (2015). Proteomics analysis of molecular risk factors in the ocular hypertensive human retina. Invest Ophthalmol Vis Sci.

[20] Ishizuka Y, Kakiya N, Witters LA, Oshiro N, Shirao T, Nawa H (2013). AMP-activated protein kinase counteracts brain-derived neurotrophic factor-induced mammalian target of rapamycin complex 1 signaling in neurons. J Neurochem.

[21] Losiewicz MK, Elghazi L, Fingar DC, Rajala RVS, Lentz SI, Fort PE (2020). mTORC1 and mTORC2 expression in inner retinal neurons and glial cells. Exp Eye Res.

[22] Pan S, Zhan S, Pan Y, Liu W, Bian L, Sun B (2015). Tetraspanin 8-rictor-integrin α3 complex is required for glioma cell migration. Int J Mol Sci.

[23] Cho JH, Kim E, Son Y, Lee D, Park YS, Choi JH (2020). CD9 induces cellular senescence and aggravates atherosclerotic plaque formation. Cell Death Differ.

[24] Zismanov V, Drucker L, Attar-Schneider O, Matalon ST, Pasmanik-Chor M, Lishner M (2012). Tetraspanins stimulate protein synthesis in myeloma cell lines. J Cell Biochem.

[25] Yang XD, Sun SC (2015). Targeting signaling factors for degradation, an emerging mechanism for TRAF functions. Immunol Rev.

[26] Dhillon B, Aleithan F, Abdul-Sater Z, Abdul-Sater AA (2019). The evolving role of TRAFs in mediating inflammatory responses. Front Immunol.

[27] Silke J, Brink R (2010). Regulation of TNFRSF and innate immune signalling complexes by TRAFs and cIAPs. Cell Death Differ.

[28] Ikeda F, Crosetto N, Dikic I (2010). What determines the specificity and outcomes of ubiquitin signaling?. Cell.

[29] Mukhopadhyay D, Riezman H (2007). Proteasome-independent functions of ubiquitin in endocytosis and signaling. Science.

[30] Linares JF, Duran A, Yajima T, Pasparakis M, Moscat J, Diaz-Meco MT (2013). K63 polyubiquitination and activation of mTOR by the p62-TRAF6 complex in nutrient-activated cells. Mol Cell.

[31] Ghosh P, Wu M, Zhang H, Sun H (2008). mTORC1 signaling requires proteasomal function and the involvement of CUL4-DDB1 ubiquitin E3 ligase. Cell Cycle.

[32] Hussain S, Feldman AL, Das C, Ziesmer SC, Ansell SM, Galardy PJ (2013). Ubiquitin hydrolase UCH-L1 destabilizes mTOR complex 1 by antagonizing DDB1-CUL4-mediated ubiquitination of raptor. Mol Cell Biol.

[33] Chidlow G, Ebneter A, Wood JP, Casson RJ (2011). The optic nerve head is the site of axonal transport disruption, axonal cytoskeleton damage, and putative axonal regeneration failure in a rat model of glaucoma. Acta Neuropathol.

[34] Kalesnykas G, Oglesby EN, Zack DJ, Cone FE, Steinhart MR, Tian J (2012). Retinal ganglion cell morphology after optic nerve crush and experimental glaucoma. Invest Ophthalmol Vis Sci.

[35] Saggu SK, Chotaliya HP, Blumbergs PC, Casson RJ (2010). Wallerian-like axonal degeneration in the optic nerve after excitotoxic retinal insult: an ultrastructural study. BMC Neurosci.

[36] Payne SC, Bartlett CA, Harvey AR, Dunlop SA, Fitzgerald M (2012). Myelin sheath decompaction, axon swelling, and functional loss during chronic secondary degeneration in rat optic nerve. Invest Ophthalmol Vis Sci.

[37] Ito Y, Shimazawa M, Tsuruma K, Mayama C, Ishii K, Onoe H (2012). Induction of amyloid-β (1-42) in the retina and optic nerve head of chronic ocular hypertensive monkeys. Mol Vis.

[38] You Y, Joseph C, Wang C, Gupta V, Liu S, Yiannikas C (2019). Demyelination precedes axonal loss in the transneuronal spread of human neurodegenerative disease. Brain.

[39] Kipfer-Kauer A, McKinnon SJ, Frueh BE, Goldblum D (2010). Distribution of amyloid precursor protein and amyloid-beta in ocular hypertensive C57BL/6 mouse eyes. Curr Eye Res.

[40] Lu Y, Brommer B, Tian X, Krishnan A, Meer M, Wang C (2020). Reprogramming to recover youthful epigenetic information and restore vision. Nature.

[41] Fang C, Bourdette D, Banker G (2012). Oxidative stress inhibits axonal transport: implications for neurodegenerative diseases. Mol Neurodegener.

[42] Roska B, Sahel JA (2018). Restoring vision. Nature.

[43] Vidal-Sanz M, Galindo-Romero C, Valiente-Soriano FJ, Nadal-Nicolás FM, Ortin-Martinez A, Rovere G (2017). Shared and differential retinal responses against optic nerve injury and ocular hypertension. Front Neurosci.

[44] Senft D, Qi J, Ronai ZA (2018). Ubiquitin ligases in oncogenic transformation and cancer therapy. Nat Rev Cancer.

[45] Jiang Y, Su S, Zhang Y, Qian J, Liu P (2019). Control of mTOR signaling by ubiquitin. Oncogene.

[46] Dengler-Crish CM, Smith MA, Inman DM, Wilson GN, Young JW, Crish SD (2014). Anterograde transport blockade precedes deficits in retrograde transport in the visual projection of the DBA/2J mouse model of glaucoma. Front Neurosci.

[47] Buckingham BP, Inman DM, Lambert W, Oglesby E, Calkins DJ, Steele MR (2008). Progressive ganglion cell degeneration precedes neuronal loss in a mouse model of glaucoma. J Neurosci.

[48] Bond WS, Hines-Beard J, GoldenMerry YL, Davis M, Farooque A, Sappington RM (2016). Virus-mediated EpoR76E therapy slows optic nerve axonopathy in experimental glaucoma. Mol Ther.

[49] Maddineni P, Kasetti RB, Patel PD, Millar JC, Kiehlbauch C, Clark AF (2020). CNS axonal degeneration and transport deficits at the optic nerve head precede structural and functional loss of retinal ganglion cells in a mouse model of glaucoma. Mol Neurodegener.

[50] Wang X, Li Q, Liu C, Hall PA, Jiang J, Katchis CD (2018). Lin28 signaling supports mammalian PNS and CNS axon regeneration. Cell Rep.

[51] Berry M, Ahmed Z, Morgan-Warren P, Fulton D, Logan A (2016). Prospects for mTOR-mediated functional repair after central nervous system trauma. Neurobiol Dis.

[52] Puttagunta R, Tedeschi A, Sória MG, Hervera A, Lindner R, Rathore KI (2014). PCAF-dependent epigenetic changes promote axonal regeneration in the central nervous system. Nat Commun.

[53] Zhou B, Yu P, Lin MY, Sun T, Chen Y, Sheng ZH (2016). Facilitation of axon regeneration by enhancing mitochondrial transport and rescuing energy deficits. J Cell Biol.

[54] Xiong X, Wang X, Ewanek R, Bhat P, Diantonio A, Collins CA (2010). Protein turnover of the Wallenda/DLK kinase regulates a retrograde response to axonal injury. J Cell Biol.

[55] Eva R, Crisp S, Marland JRK, Norman JC, Kanamarlapudi V, ffrench-Constant C (2012). ARF6 directs axon transport and traffic of integrins and regulates axon growth in adult DRG neurons. J Neurosci.

[56] Park KK, Liu K, Hu Y, Smith PD, Wang C, Cai B (2008). Promoting axon regeneration in the adult CNS by modulation of the PTEN/mTOR pathway. Science..

[57] Pita-Thomas W, Mahar M, Joshi A, Gan D, Cavalli V (2019). HDAC5 promotes optic nerve regeneration by activating the mTOR pathway. Exp Neurol.

[58] Li S, Yang C, Zhang L, Gao X, Wang X, Liu W (2016). Promoting axon regeneration in the adult CNS by modulation of the melanopsin/GPCR signaling. Proc Natl Acad Sci USA.

[59] Duan X, Qiao M, Bei F, Kim IJ, He Z, Sanes JR (2015). Subtype-specific regeneration of retinal ganglion cells following axotomy: effects of osteopontin and mTOR signaling. Neuron..

[60] Yao A, van Wijngaarden P (2020). Metabolic pathways in context: mTOR signalling in the retina and optic nerve—A review. Clin Exp Ophthalmol.

[61] Munemasa Y, Kitaoka Y (2015). Autophagy in axonal degeneration in glaucomatous optic neuropathy. Prog Retin Eye Res.

[62] Russo R, Nucci C, Corasaniti MT, Bagetta G, Morrone LA (2015). Autophagy dysregulation and the fate of retinal ganglion cells in glaucomatous optic neuropathy. Prog Brain Res.

[63] Rodríguez-Muela N, Germain F, Mariño G, Fitze PS, Boya P (2012). Autophagy promotes survival of retinal ganglion cells after optic nerve axotomy in mice. Cell Death Differ.

[64] Su W, Li Z, Jia Y, Zhuo Y. Rapamycin is neuroprotective in a rat chronic hypertensive glaucoma model [published correction appears in PLoS ONE. 2019 Mar 4;14: e0213489]. PLoS ONE. 2014;9:e99719. 10.1371/journal.pone.0099719 PMC4055719 24923557

[65] Knoferle J, Koch JC, Ostendorf T, Michel U, Planchamp V, Vutova P (2010). Mechanisms of acute axonal degeneration in the optic nerve in vivo. Proc Natl Acad Sci USA.

[66] Park HY, Kim JH, Park CK (2012). Activation of autophagy induces retinal ganglion cell death in a chronic hypertensive glaucoma model. Cell Death Dis.

[67] Wang B, Jie Z, Joo D, Ordureau A, Liu P, Gan W (2017). TRAF2 and OTUD7B govern a ubiquitin-dependent switch that regulates mTORC2 signalling. Nature..

[68] Tonoli H, Barrett JC (2005). CD82 metastasis suppressor gene: a potential target for new therapeutics?. Trends Mol Med.

[69] Florin L, Lang T (2018). Tetraspanin assemblies in virus infection. Front Immunol.

[70] Noy PJ, Yang J, Reyat JS, Matthews AL, Charlton AE, Furmston J (2016). TspanC8 tetraspanins and A disintegrin and metalloprotease 10 (ADAM10) interact via their extracellular regions: Evidence for distinct binding mechanisms for different TspanC8 proteins. J Biol Chem.

[71] Seipold L, Altmeppen H, Koudelka T, Tholey A, Kasparek P, Sedlacek R (2018). In vivo regulation of the A disintegrin and metalloproteinase 10 (ADAM10) by the tetraspanin 15. Cell Mol Life Sci.

[72] Murru L, Moretto E, Martano G, Passafaro M (2018). Tetraspanins shape the synapse. Mol Cell Neurosci.

[73] Stuck MW, Conley SM, Naash MI (2016). PRPH2/RDS and ROM-1: Historical context, current views, and future considerations. Prog Retin Eye Res.

[74] Chi W, Li F, Chen H, Wang Y, Zhu Y, Yang X (2014). Caspase-8 promotes NLRP1/NLRP3 inflammasome activation and IL-1β production in acute glaucoma. Proc Natl Acad Sci USA.

[75] Alavi MV, Bette S, Schimpf S, Schuettauf F, Schraermeyer U, Wehrl HF (2007). A splice site mutation in the murine Opa1 gene features pathology of autosomal dominant optic atrophy. Brain.

[76] Kretschmer F, Kretschmer V, Kunze VP, Kretzberg J (2013). OMR-arena: automated measurement and stimulation system to determine mouse visual thresholds based on optomotor responses. PLoS ONE.

[77] Li X, Zheng L, Xia Q, Liu L, Mao M, Zhou H (2019). A novel cell-penetrating peptide protects against neuron apoptosis after cerebral ischemia by inhibiting the nuclear translocation of annexin A1. Cell Death Differ.

[78] Lopes FM, da Motta LL, De Bastiani MA, Pfaffenseller B, Aguiar BW, de Souza LF (2017). RA differentiation enhances dopaminergic features, changes redox parameters, and increases dopamine transporter dependency in 6-hydroxydopamine-induced neurotoxicity in SH-SY5Y cells. Neurotox Res.

[79] Hu Y, Vinayagam A, Nand A, Comjean A, Chung V, Hao T (2018). Molecular Interaction Search Tool (MIST): an integrated resource for mining gene and protein interaction data. Nucleic Acids Res.

[80] Shannon P, Markiel A, Ozier O, Baliga NS, Wang JT, Ramage D (2003). Cytoscape: a software environment for integrated models of biomolecular interaction networks. Genome Res.

