# Supplementary material for: CD82 protects against glaucomatous axonal transport deficits via mTORC1 activation in mice
Source: Cell Death Dis. 2021 Dec 11;12(12):1149. doi: 10.1038/s41419-021-04445-6 (PMC8665930; doi:10.1038/s41419-021-04445-6)
Supplement: Supplementary file 2 — Suplemental Information [file 41419_2021_4445_MOESM2_ESM.pdf]

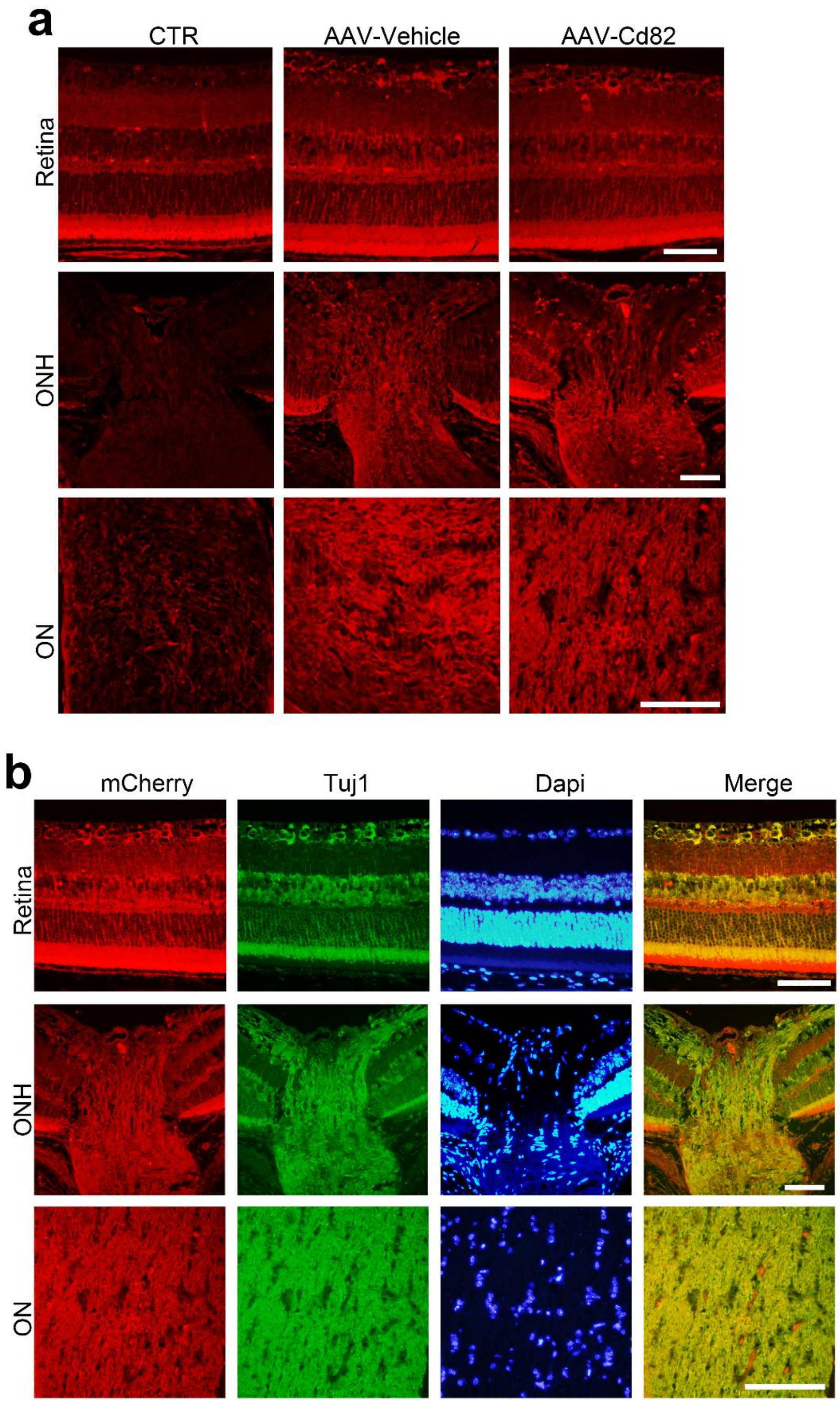

Figure S1. AAV-Cd82 transfection in retina, ON and ONH. (a) Immunostaining of AAV tag mCherry shown in retina, ON and ONH in CTR, AAV-Vehicle, AAV-Cd82 groups 28 days after virus injection, n=5 eyes. Scale bar, 50μm. **(b)** Co-immunofluorescent staining of mCherry (red) with RGC marker Tuj1 (green) in AAV-Cd82 group 28 days after virus injection, n=5 eyes. Scale bar, 50μm.

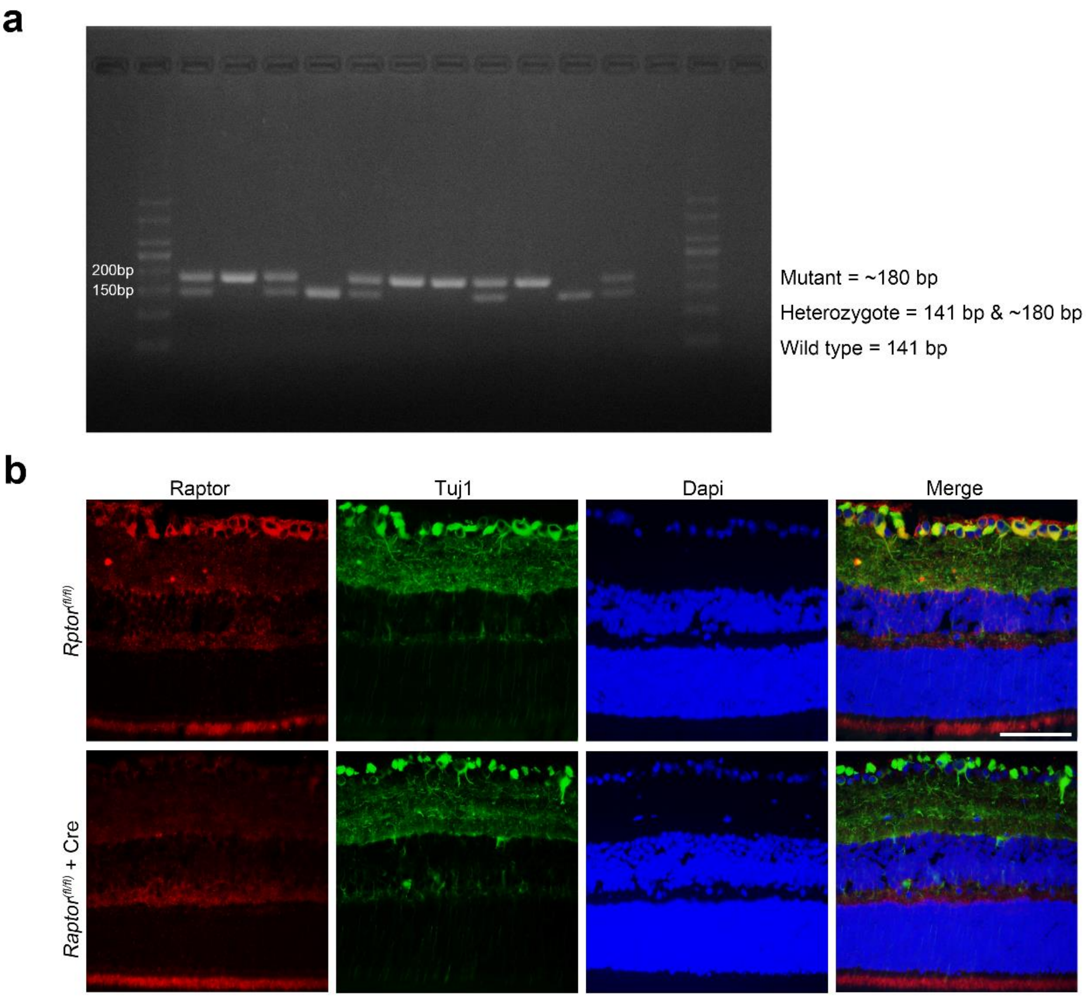

Figure S2. Knockdown of Raptor in *Rptor*$^{fl/fl}$ mouse. (a) Genotyping of wild type, *Rptor*$^{fl/+}$ and *Rptor*$^{fl/fl}$ mice by agarose gel electrophoresis. **(b)** Co-immunofluorescent staining of Raptor (red) with RGC marker Tuj1 (green) in retina sections of *Rptor*$^{fl/fl}$ mice with or without knockdown of Raptor by Cre. Scale bar, 50μm.
